# CSGDN: contrastive signed graph diffusion network for predicting crop gene–phenotype associations

**DOI:** 10.1093/bib/bbaf062

**Published:** 2025-02-20

**Authors:** Yiru Pan, Xingyu Ji, Jiaqi You, Lu Li, Zhenping Liu, Xianlong Zhang, Zeyu Zhang, Maojun Wang

**Affiliations:** National Key Laboratory of Crop Genetic Improvement, Hubei Hongshan Laboratory, Huazhong Agricultural University, 430070 Hubei, China; National Key Laboratory of Crop Genetic Improvement, Hubei Hongshan Laboratory, Huazhong Agricultural University, 430070 Hubei, China; National Key Laboratory of Crop Genetic Improvement, Hubei Hongshan Laboratory, Huazhong Agricultural University, 430070 Hubei, China; National Key Laboratory of Crop Genetic Improvement, Hubei Hongshan Laboratory, Huazhong Agricultural University, 430070 Hubei, China; National Key Laboratory of Crop Genetic Improvement, Hubei Hongshan Laboratory, Huazhong Agricultural University, 430070 Hubei, China; National Key Laboratory of Crop Genetic Improvement, Hubei Hongshan Laboratory, Huazhong Agricultural University, 430070 Hubei, China; National Key Laboratory of Crop Genetic Improvement, Hubei Hongshan Laboratory, Huazhong Agricultural University, 430070 Hubei, China; National Key Laboratory of Crop Genetic Improvement, Hubei Hongshan Laboratory, Huazhong Agricultural University, 430070 Hubei, China

**Keywords:** gene–phenotype associations, graph neural networks, signed bipartite networks, signed graph neural networks

## Abstract

Positive and negative association prediction between gene and phenotype helps to illustrate the underlying mechanism of complex traits in organisms. The transcription and regulation activity of specific genes will be adjusted accordingly in different cell types, developmental timepoints, and physiological states. There are the following two problems in obtaining the positive/negative associations between gene and phenotype: (1) high-throughput DNA/RNA sequencing and phenotyping are expensive and time-consuming due to the need to process large sample sizes; (2) experiments introduce both random and systematic errors, and, meanwhile, calculations or predictions using software or models may produce noise. To address these two issues, we propose a **C**ontrastive **S**igned **G**raph **D**iffusion **N**etwork, **CSGDN**, to learn robust node representations with fewer training samples to achieve higher link prediction accuracy. CSGDN uses a signed graph diffusion method to uncover the underlying regulatory associations between genes and phenotypes. Then, stochastic perturbation strategies are used to create two views for both original and diffusive graphs. Lastly, a multiview contrastive learning paradigm loss is designed to unify the node presentations learned from the two views to resist interference and reduce noise. We perform experiments to validate the performance of CSGDN in three crop datasets: *Gossypium hirsutum*, *Brassica napus*, and *Triticum turgidum*. The results show that the proposed model outperforms state-of-the-art methods by up to 9. 28% AUC for the prediction of link sign in the *G. hirsutum* dataset. The source code of our model is available at https://github.com/Erican-Ji/CSGDN.

## Introduction

The prediction of positive/negative associations between the gene and phenotype has been known to be important in the field of biology, with broad applications in breeding crops [1]. By constructing the association between genomic variations (such as single nucleotide polymorphisms) and phenotypes within a large population, a genome-wide association study (GWAS) uncovers the genetic basis underlying the formation of phenotypes [2–5]. In various crops such as rice, maize, wheat, cotton, and rapeseed, GWAS has been widely applied to identify quantitative phenotype loci (QTL) related to yield [6–10], quality [10–13], resistance [8, 12, 14–17], and other agronomic phenotypes [9, 18, 19], and to further help identify candidate genes. In sorghum, an alkaline tolerance related gene named *Alkali Tolerance 1* (*AT1*) was located through GWAS analysis of 352 representative accessions, which encodes a heterotrimeric G protein subunit $\gamma $ (G$\gamma $) [10]. The non-functional mutant can improve the yield in sorghum, rice, and maize in alkaline soils. In rapeseed, Zhang *et al*. (2024) located a previously unknown gene on chromosome A02, named *BnaA02.SE* [8]. Overexpression of this gene contributes to longer and larger siliquae, indicating that the gene is positively associated with rapeseed yield.

GWAS can identify phenotypic-related QTLs, but the QTL sizes vary from several kilobases (kb) to megabases (Mb), which are determined by the decay distance of the linkage disequilibrium [3, 4]. This increases the difficulty in identifying candidate genes for a particular QTL. A simple candidate gene mining strategy is to initially select genes with non-synonymous mutations, followed by further screening with functional annotations and expression pattern analysis. This strategy was widely used in the research of crops such as wheat [6, 7], rapeseed [20], and cotton [10, 21]. However, this strategy has many limitations, as it focuses primarily on genes with coding sequence variations and overlooks the disturbance of gene expression level changes in the phenotype.

With the increase in association analysis, researchers have observed that the vast majority of GWAS signals are located in the noncoding regions of the genome, implying that gene expression regulation is the primary mechanism underlying phenotypic differences between individuals [22]. Various methods have been developed to integrate GWAS with transcriptome data to identify gene–phenotype associations. These methods can be broadly categorized into three types: colocalization analysis, Mendelian randomization and transcriptome-wide association studies (TWAS) [5]. Colocalization analysis typically refers to a statistical analysis that is used to determine whether genetic association signals from two traits overlap in a genomic region due to shared causal variants. A representative method is ’COLOC’, which uses approximate Bayes factors to calculate the posterior probability since a variant is causally associated with both traits [23, 24]. Mendelian randomization is a statistical method that uses one or more genetic variants as instrumental variables to examine the causal relationship between exposure and outcome. A representative method is summary data-based Mendelian randomization (SMR), which integrates summary data from GWAS and eQTL to identify genes whose expression levels are associated with traits through shared genetic effects [25]. The concept of TWAS generally refers to the analysis that associates the expression levels of genes with the target traits. A representative method is FUSION-TWAS, which constructs predictive models of gene expression and associates genetically predicted gene expression with traits, in order to address sampling difficulties or reduce sequencing costs [26].

The above three methods have been widely applied in crop research. In rapeseed, 5285 gene–trait associations were identified using TWAS, and 726 gene–trait associations were identified using COLOC [27]. In rice, 340 gene–trait associations for starch content were identified using TWAS, and 9 gene–trait associations using COLOC [28]. In maize, 97 gene–trait associations were identified for drought tolerance using SMR [29]. TWAS is capable of replicating the results from COLOC and SMR and identifies more associations. However, TWAS still relies on the association analysis framework, lacking an accurate predictive capacity for genes with low variant density.

In addition, traditional methods can introduce various types of errors and noise. For example, biases such as systematic bias, coverage bias, and batch effects often affect Next Generation Sequencing data [30]. These biases are introduced by the sequencing platform, genome content, and experimental variability [31]. The experimental design and the selection of sample replication depend on the specificity of the species, which to some extent affects the bias of the quantitative results [32]. The accurate TWAS results [33] also depend on the high quality of the training data. Therefore, it is crucial to develop models to predict positive/negative gene–phenotype associations, minimizing the biases and errors produced in traditional experimental methods.

As mentioned, there are two major issues in predicting positive/negative regulation associations between gene and phenotype:

(1) **The high cost associated with large sample sizes.** Traditional methods such as GWAS and TWAS require large sample sizes to detect associations, increasing the costs of sequencing and data analysis. Larger samples also require more time and resources, making the process expensive and slow.
(2) **Data noise from experiments and methods.** Experiments often introduce biases such as systematic errors and batch effects. Noise makes it difficult to accurately detect the associations between gene and phenotype.

**Figure 1:**
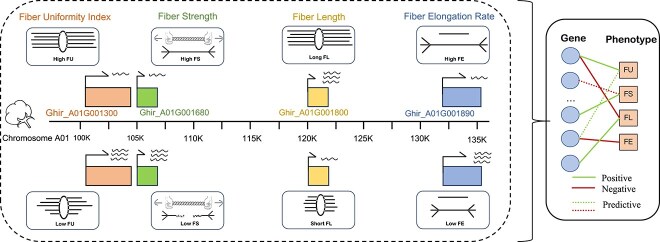
CSGDN abstracts the associations between genes and phenotypes into a signed bipartite graph. Our task is to predict the gene associations by constructing a neural network framework for the bipartite graph.

In response to these two issues, we propose a **C**ontrastive **S**igned **G**raph **D**iffusion **N**etwork, **CSGDN** to solve these issues of predicting the positive/negative regulation associations. In this approach, as shown in the Fig. 1, gene–phenotype associations are modeled as a signed bipartite graph, where gene and phenotype nodes are connected by either positive or negative edges, indicating positive/negative regulation of genes in phenotypes. **For obstacle 1**, we apply a signed graph diffusion theory to uncover hidden associations between genes and phenotypes. The diffusion method helps address the challenge of large sample size requirements by capturing complex associations through a smaller dataset, reducing the overall cost. **For obstacle 2**, we employ stochastic perturbation techniques to generate two views of both the original and diffused graphs. A multiview contrastive learning loss unifies the node representations from both views, helping to reduce interference and noise. In summary, our model effectively addresses the cost and noise challenges as mentioned. CSGDN can predict gene–phenotype associations across crop species using only small-sample associations and shows strong robustness against interference from experimental noise.

To evaluate the effectiveness of CSGDN, we conduct extensive experiments on three crop datasets: *Gossypium hirsutum*, *Brassica napus*, and *Triticum turgidum*. Our results demonstrate that CSGDN consistently outperforms baseline models from both unsigned GNNs and signed GNNs. For unsigned GNNs (GCN, GAT, GRACE), CSGDN achieves improvements in AUC of up to 6.66%, 7.13%, and 7.26%, respectively. For signed GNNs (SGCN, SGCL, SGNNMD), CSGDN outperforms baselines with AUC gains of 5.96%, 5.29%, and 9.29%, respectively. For methods in the field of gene–phenotype association prediction, we also compare our model with them (XGDAG, ModulePred), which also show a better result. These results underscore the superior ability of CSGDN to enhance link sign prediction tasks in diverse crop datasets. We randomly sample 80% of the datasets to evaluate CSGDN’s performance in addressing the challenge of small sample sizes. The results show that our model outperforms the baselines on AUC, F1, Micro, Macro, AUPR, and Precision with improvements of up to approximately 10% across all metrics. Specifically, CSGDN improves F1 by up to 9.51% with 10% perturbed edges and improves Micro-F1 by up to 12.64% with 20% perturbed edges compared to SGNNMD. These experiments clearly highlight the effectiveness of our model CSGDN.

Overall, our contributions are summarized as follows:

- We propose a model for the prediction of the gene–phenotype association, which outperforms the baselines in terms of link-sign prediction performance.
- By combining the diffusion method and the contrastive learning framework, our model effectively addresses the challenges of high cost and noise in positive/negative regulation association prediction.

## Related work

**Association prediction**. Recently, significant progress has been made in the field of association prediction. SGNNMD proposed by Zhang *et al*. [34], extracts subgraphs around miRNA-disease pairs from signed bipartite networks and uses biological features to accurately predict deregulation types of miRNA-disease associations. The framework of HGATLDA proposed by Zhao *et al*. [35], effectively integrates meta-path-based heterogeneous graphs and attention mechanisms to improve the prediction of lncRNA–disease associations, addressing limitations in feature fusion, complex associations, and data imbalance. MTRD proposed by Zhang *et al*. [36] integrates multi-scale topology embeddings and node attributes with advanced learning mechanisms, achieving superior performance in predicting drug-disease associations and effectively identifying potential candidate diseases for specific drugs. BGF-CMAP proposed by Guo *et al*. [37] integrates advanced techniques like Word2vec and graph embedding algorithms to extract both sequence and interaction features, significantly enhancing the prediction of complex circRNA–miRNA associations. MDGF-MCEC, proposed by Wu *et al*. [38], utilizes a multi-view dual attention GCN and cooperative ensemble learning to predict circRNA-disease associations, achieving enhanced feature representation and classification through multi-similarity relation graphs and attention-based feature adjustment. Although neural networks have shown great potential in the association prediction fields, there is still insufficient progress in developing direct prediction methods for gene–phenotype associations, particularly due to high costs associated with large sample sizes and the noise introduced throughout the experimental processes.

**Signed Graph Neural Networks**. Recently, neural approaches have gained traction in signed graph representation, we refer to such methods collectively as Signed Graph Neural Networks (SGNNs) [39–42]. With innovations like SiNE [43] pioneering deep learning use using triangle structures with mixed edge polarities. SiNE optimizes an objective grounded in balance theory for embedding generation. The advent of SGCN [39] extended GCN’s [44] scope to signed graphs, incorporating balance theory for multi-hop relationship discernment. Models like SiGAT [45], SNEA [46], SDGNN [47], and SGCL [48], rooted in attention mechanisms [49], further enrich this landscape.

Beyond the aforementioned approaches, some SGNNs claim to possess robustness against noise. SGCL [48] first adopts the contrastive learning paradigm in signed graph analysis, which can learn invariant representation of nodes under minor random perturbation and help to enhance the robustness of the model. RSGNN [50] is another trial to enhance the robustness of SGNNs. It enables the model to learn a cleaner structure, less influenced by noise. However, considering the substantial computational overhead involved in learning a new adjacency matrix, this approach is only feasible for smaller node sets. Given the vast number of gene types, this method is impractical for gene–phenotype datasets.

## Materials

### Datasets

We use datasets from three species including *G. hirsutum* [11], *B. napus* [19], and *T. turgidum* [51] to input our model. For each species data, we perform the TWAS process method to obtain associations between gene and phenotype. Initially, phenotype data are standardized using the qqnorm function in R and principal component analysis is conducted for population structure data using TASSEL software [52]. FaST-LMM software [53] is used to perform GWAS, considering phenotype data and population structure. We use Fusion software to perform TWAS [33] to obtain TWAS $z score$ of associations between partial genes and phenotypes. The positive or negative sign of TWAS $z score$ indicates positive/negative regulation, respectively. Note that the TWAS process can only calculate partial associations between genes and phenotypes. We separate the genes that can be associated with TWAS from those that cannot be calculated by TWAS within the species and input them separately into the model.

***Gossypium hirsutum*** [11]. The phenotype data include four phenotypes at five fiber developmental time points, including 4 DPA (days post anthesis), 8 DPA, 12 DPA, 16 DPA, and 20 DPA. The four phenotypes are Fiber length (FL), Fiber strength (FS), Fiber elongation rate (FE), and Fiber Uniformity (FU), respectively. The reference genome is from Wang *et al*. [54]. The result of the TWAS process contains 523 associations between genes and the phenotype FL, 1129 associations between genes and FS, 1521 associations between genes and FE, 509 associations between genes and FU.

***Brassica napus*** [19]. *B. napus* dataset includes 20 DPA and 40 DPA. The type of phenotype is only the seed oil content (SOC). The reference genome (*B. napus* v4.1) can be downloaded from Genoscope (http://www.genoscope.cns.fr/brassicanapus/). The number of associations between genes in *B. napus* and SOC is 605 at 20 DPA and 148 at 40 DPA, separately. We consider the different timepoints to be distinct phenotypes.

***Triticum turgidum*** [51]. *T. turgidum* dataset includes four phenotypes: Biomass (BM), Root length (RL), Root area (RA), and Root volume (RV). We use WEW v2.1 as the reference genome [55]. The result of the TWAS process includes 36 associations with these four phenotypes, with BM having 1 association, RA having 2, RL having 17, and RV having 15.

### Gene sequences similarity

As complement to our model, we calculate the similarity between gene sequences as features. The software BLAST+/2.9.0 [56] is used to align sequences between gene pairs. BLAST can calculate similarity scores between pairs of genes. We use BLAST+ 2.9.0 [56] to obtain a similarity matrix $S \in \mathbb{R}^{n \times n}$, where $n$ = $|\mathcal{V}|$. We use the similarity matrix $S$ as the initial feature input for genes. To be specific, the input feature $h_{i}^{(0)}$ of gene $v_{i}$ is the $i$th row of the similarity matrix.

## Preliminary

We construct a signed bipartite graph $\mathcal{G} = \{\mathcal{V},\mathcal{U}, \mathcal{\mathcal{E}}^{+}, \mathcal{E}^{-}\}$, where a matrix set of gene $\mathcal{V} = \{v_{1}, v_{2}, \ldots , v_{|\mathcal{V}|}\}$ and a set of phenotypes $\mathcal{U} = \{u_{1}, u_{2}, \ldots , u_{|\mathcal{U}|}\}$ are given. The edge set $\mathcal{E}$ represents deregulation associations, where $\mathcal{E} = \mathcal{E}^{+} \cup \mathcal{E}^{-}$ and $\mathcal{E}^{+} \cap \mathcal{E}^{-} = \emptyset $. Each edge belongs to one regulation type: a positive edge $(v, u) \in \mathcal{E}^{+}$ indicates that the specific gene up-regulates the phenotype, while a negative edge $(v, u) \in \mathcal{E}^{-}$ represents down-regulation.

We define a subset of the edge set $\mathcal{E}$ as $\mathcal{E}_{\text{TWAS}}$, which consists of edges that can be calculated using TWAS processes. Consequently, the node set $\mathcal{V}_{\text{TWAS}}$ is defined as the set of genes that correspond to the edges in $\mathcal{E}_{\text{TWAS}}$: $\mathcal{E}_{\text{TWAS}} \subseteq \mathcal{E} \quad \text{such that} \quad \forall (v, u) \in \mathcal{E}_{\text{TWAS}}$, where $v \in \mathcal{V}, u \in \mathcal{U}$. The set of genes associated with TWAS can then be expressed as follows: $\mathcal{V}_{\text{TWAS}} = \{ v \in \mathcal{V} \,|\, \exists u \in \mathcal{U}, \ (v, u) \in \mathcal{E}_{\text{TWAS}} \}$. The number of nodes is defined as $\mathcal{N}$.

Given $\mathcal{G} = \{\mathcal{U}, \mathcal{V}, \mathcal{\mathcal{E}}^{+}, \mathcal{E}^{-}\}$, the goal is to learn a function $f$ to map nodes $v_{i} \in \mathcal{V}$ and $u_{j} \in \mathcal{U}$ to low-dimensional embeddings $z_{v_{i}} \in \mathbb{R}^{d}$ and $z_{u_{j}} \in \mathbb{R}^{d}$, where $d$ is the dimension of node embeddings. These embeddings should be useful for downstream tasks such as link sign prediction. This setup forms a crucial framework for studying the associations between genes and phenotypes, allowing for the prediction of up-regulation or down-regulation patterns between genes and phenotypes.

All the notations used throughout this paper are shown in the Table 1.

**Table 1:** Main notations used throughout this paper

**Notations**	**Descriptions**
$\mathcal{G}$	A signed graph
$\mathcal{V}$	A set of gene nodes
$\mathcal{U}$	A set of phenotype nodes
$\mathcal{E}$	A set of edges, where $\mathcal{E}^{+}$ denotes positive edges and $\mathcal{E}^{-}$ denotes negative edges
$\mathcal{E}_{\text{TWAS}}$	A subset of edges calculated through TWAS processes, defined as $\mathcal{E}_{\text{TWAS}} \subseteq \mathcal{E}$ such that $\forall (v, u) \in \mathcal{E}_{\text{TWAS}}$, where $v \in \mathcal{V}$ and $u \in \mathcal{U}$
$\mathcal{V}_{\text{TWAS}}$	The set of gene nodes associated with TWAS, defined as $\mathcal{V}_{\text{TWAS}} = \{ v \in \mathcal{V} \,|\, \exists u \in \mathcal{U}, \ (v, u) \in \mathcal{E}_{\text{TWAS}} \}$
$\mathcal{S}$	A similarity matrix of genes
$\mathbf{A}$	The adjacency matrix of the graph $\mathcal{G}$
$\mathbf{D}$	The outdegree diagonal matrix of the graph $\mathcal{G}$
$\mathbf{G_{k}}$	The graphs obtained by the graph augmentation method
$h_{i,k}^{(l)}$	The representation of the $i$th node for the $l$th layer in the $k$th augmented graph
$z_{i,k}^{+}$	The final representation of the $i$th node in the positive graph of the $k$th augmented graph
$z_{i,k}^{-}$	The final representation of the $i$th node in the negative graph of the $k$th augmented graph

## Proposed method

In this section, we introduce the **CSGDN** to tackle the aforementioned challenges, including enhancing structural information for small samples to reduce the expense in TWAS analysis and reducing noise from the whole process. As mentioned in the MATERIALS section, we divide genes in each species into two parts: one part is associated with phenotypes through the TWAS process, and the other part lacks such associations.

**Figure 2:**
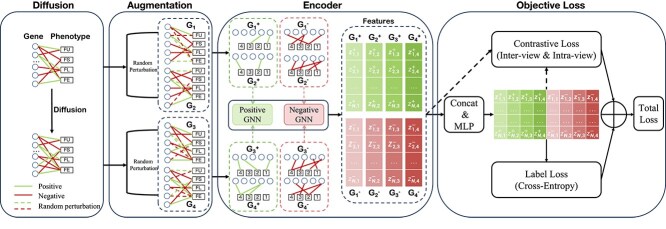
The overall architecture of CSGDN. The figure is divided into four main parts: (1) the graph diffusion processes, which generates a diffusion graph $\mathcal{S}$, (2) the signed graph augmentation, which generates four augmented graphs, (3) the encoder, which processes the augmented graph and generates feature representations, and (4) the objective losses, which define the optimization goal for the model. These components work together to enable accurate gene–phenotype association prediction.

The main framework of the proposed CSGDN model is to encode genes associated with TWAS and phenotypes, which is illustrated in Fig. 2. The main framework includes four key components: (1) graph diffusion, (2) graph augmentation, (3) the encoder, and (4) the objective loss. To be specific, we first use a graph diffusion method to uncover the potential relationships between genes and phenotypes, resulting in a diffusion graph. Then, we randomly remove edges from the original graph and the diffusion graph to obtain augmented graphs. After encoding these augmented graphs with the encoder, we obtain node representations to calculate the contrastive loss.

In addition, we train an MLP to learn the encoding capability of the model’s main framework for TWAS genes, in order to encode genes that lack TWAS associations.

### Signed graph diffusion

The collection of large-scale crop sample data requires a high cost. Therefore, it is necessary to develop methods that can maintain high-precision prediction even with a low number of training samples. In the field of biology, the effects of genes on phenotypes involve both positive and negative regulation, meaning that genes can promote or inhibit phenotypes through complex mechanisms. This forms a bipartite signed graph which consists of positive/negative edge types and two disjoint sets of nodes. In situations where crop sample data are scarce, the resulting signed graph structure is relatively sparse.

Traditional ranking models, such as PageRank [57], can mine the potential relationships between nodes to increase the number of edges, but are only suitable for graphs with a single type of positive edges. Wu *et al*. [58] proposed Troll-Trust model, which is a variant of PageRank, without explaining the complex relationships between the negative and positive edges. To better handle the signed graph with two edge types, we use a diffusion operation based on the Signed Random Walk with Restart (SRWR) algorithm proposed by previous researchers [59] to uncover the potential associations between genes and phenotypes, obtaining a diffusion graph. The diffusion graph provides richer structural information, which can effectively alleviate the problem of sparsity in graph data, while also providing a new graph structure for subsequent contrastive learning operations.

We introduce a signed random surfer for a bipartite signed graph. The surfer randomly surfs between nodes and traverses their relationships. When the surfer encounters a positive edge, it maintains a positive sign +; when it encounters a negative edge, it flips its sign to negative −. Initially, the surfer carries a positive sign + at node $ u $. If the surfer is at node $ v $ at this time and the restart probability is $ c $, the probability that the surfer randomly surfs to neighboring nodes is $ 1 - c $.

Based on the SRWR algorithm [59], we can define the adjacency matrix $ \mathbf{A} $ of the signed graph $\mathcal{G}$. $ \mathbf{D} $ is the out-degree diagonal matrix, where $ \mathbf{D}_{ii} = \sum _{j} |\mathbf{A}|_{ij} $. The semi-row normalized matrix is $ \tilde{\mathbf{A}} = \mathbf{D}^{-1} \mathbf{A} $. We decompose $ \tilde{\mathbf{A}} $ into two matrices: a positive semi-row normalized matrix ($ \tilde{\mathbf{A}}_{+} $) and a negative semi-row normalized matrix ($ \tilde{\mathbf{A}}_{-} $), such that $ \tilde{\mathbf{A}} = \tilde{\mathbf{A}}_{+} - \tilde{\mathbf{A}}_{-} $. We formulate the probability vectors, $\mathbf{r}^{+}$ and $\mathbf{r}^{-}$.

Since simple balance theory with four types of relationships cannot explain complex imbalanced relationships, introducing balance attenuation factors $ \beta $ and $ \gamma $ is beneficial. When a negative walker encounters a negative edge at a node, its sign will switch to positive with probability $ \beta $, or remain negative with probability $ 1 - \beta $. Similarly, when the negative walker encounters a positive edge at node $ m $, its sign will remain negative with probability $ \gamma $, or switch to positive with probability $ 1 - \gamma $. The diffusion model with balance attenuation factors becomes


(1) \begin{align*} \mathbf{r}^{+} &= (1 - c) \left(\tilde{\mathbf{A}}_{+}^{T} \mathbf{r}^{+} + \beta \tilde{\mathbf{A}}_{-}^{T} \mathbf{r}^{-} + (1 - \gamma) \tilde{\mathbf{A}}_{+}^{T} \mathbf{r}^{-}\right) + c\mathbf{q} \nonumber \\ \mathbf{r}^{-} &= (1 - c) \left(\tilde{\mathbf{A}}_{-}^{T} \mathbf{r}^{+} + \gamma \tilde{\mathbf{A}}_{+}^{T} \mathbf{r}^{-} + (1 - \beta) \tilde{\mathbf{A}}_{-}^{T} \mathbf{r}^{-}\right) \end{align*}

where $ \tilde{\mathbf{A}}_{+}^{T} $ and $ \tilde{\mathbf{A}}_{-}^{T} $ are the transposed adjacency matrices for positive and negative edges, respectively. $\mathbf{q}$ is the seed node vector at node $ s $. Initially, set $ \mathbf{r}^{+} = \mathbf{q} $, $\mathbf{r}^{-} = 0 $, and define $$ \mathbf{r}^{\prime} = \begin{bmatrix}\mathbf{r}^{+} \\ \mathbf{r}^{-} \end{bmatrix}^{T} $$.

By repeated iterative computation of $ \mathbf{r}^{+} $ and $ \mathbf{r}^{-} $, we can concatenate $ \mathbf{r}^{+} $ and $ \mathbf{r}^{-} $ into $$ \mathbf{r} = \begin{bmatrix} \mathbf{r}^{+} \\ \mathbf{r}^{-} \end{bmatrix}^{T} $$. Then by computing the error $ \delta $ between the current iteration result $ \mathbf{r} $ and the previous iteration result $ \mathbf{r}^{\prime} $, where $\delta = || \mathbf{r}- \mathbf{r}^{\prime}||$, we update $ \mathbf{r} $ for the next iteration. The iteration stops when the error $ \delta $ is smaller than a tolerance $\epsilon $.

For each node, the algorithm return an $ \mathbf{r}^{+} $ and an $ \mathbf{r}^{-} $. We combine all $ r^{+} $ and $ r^{-} $ into a positive matrix $ \mathbf{r_{p}} \in \mathbb{R}^{n \times n} $ and a negative matrix $ \mathbf{r_{n}} \in \mathbb{R}^{n \times n} $. Note that the relationship between edges ($ u \to v $) and ($ v \to u $) may differ, and the sign may even be opposite. This means $ \mathbf{r_{p}}(u,v) \ne \mathbf{r_{p}}(v,u)$ may occur.

To address this and generate an undirected graph for subsequent process, we transpose $ \mathbf{r_{p}} $ and $ \mathbf{r_{n}} $ to $ \mathbf{r_{p}}^{T} $ and $ \mathbf{r_{n}}^{T} $ respectively where $ \mathbf{r_{p}}^{T}(u,v) = \mathbf{r_{p}}(v,u) $ ( or $ \mathbf{r_{n}}^{T}(u,v) = \mathbf{r_{n}}(v,u) $). For each node, we take the maximum value between $ \mathbf{r_{p}} $ and $ \mathbf{r_{p}}^{T} $ to form $ \mathbf{r_{p}}_{\text{max}} $ and $ \mathbf{r_{n}}_{\text{max}} $. Then we calculate $ \mathbf{r_{p}}_{\text{max}} - \mathbf{r_{n}}_{\text{max}}$ for each node at the corresponding position to generate the symmetric $ \mathbf{r_{d}} $ matrix, which can also be called the diffuse matrix $\mathcal{S}$.




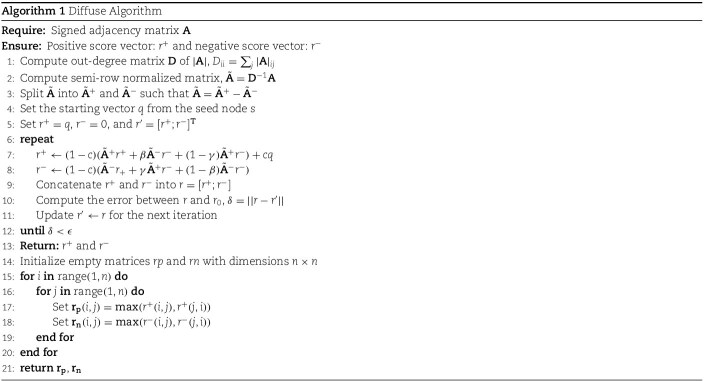



### Graph augmentation

Generating different views is crucial in contrastive learning methods. In this study, we primarily focus on randomly removing edges on both the original graph and the diffusion graph to obtain different views. For each graph, we can construct a matrix $\mathcal{M} \in \mathbb{R}^{2 \times n} $, where each column represents one edge (such as u-¿v) containing only existing edges. Then we generate a random masking matrix $\widetilde{\mathcal{R}} \in \mathbb{R}^{1 \times n}$ drawn from a uniform distribution over $[0,1]$, denoted as $\widetilde{\mathcal{R}}_{i} \sim \text{Uniform}(0,1)$. Setting a threshold $p_{r} =0.1$, we reset $\widetilde{\mathcal{R}}_{i}=0$ to denote the deletion of the corresponding edge if $\widetilde{\mathcal{R}}_{i} < 0.1$. The resulting matrix can be computed as follows: $\widetilde{\mathcal{M}}=\mathcal{M} \circ \widetilde{\mathcal{R}}$, where $ (x \circ y)_{i} = x_{i} y_{i} $ is Hadamard product.

We perform this process twice on both the original graph $\mathcal{G}$ and the diffusion graph $\mathcal{S}$ separately, randomly removing 10% of the edges each time. Therefore, we can obtain four augmented graphs, denoted as $\mathbf{G}_{1}$, $\mathbf{G}_{2}$, $\mathbf{G}_{3}$, and $\mathbf{G}_{4}$, respectively.

### Graph encoder

After data augmentation, we obtain four augmented graphs including $\mathbf{G}_{1}$, $\mathbf{G}_{2}$, $\mathbf{G}_{3}$, and $\mathbf{G}_{4}$. For convenience, we define the set of augmented graphs as $\mathbf{G}_{k}=\left \{ \mathbf{G}_{1},\mathbf{G}_{2},\mathbf{G}_{3},\mathbf{G}_{4} \right \}$, where k=1,2,3,4. Edge types are classified into positive and negative, meaning the two types of effects of genes on specific phenotypes, namely up-regulation and down-regulation. Consequently, it is imperative to design two distinct GNNs to separately aggregate information from positive and negative neighbors. We split each graph into two graphs containing only positive or negative edges, where $\mathbf{G}_{i}^{+}=(\mathcal{U} \cup \mathcal{V}, \mathcal{E}^{+})$ and $\mathbf{G}_{i}^{-}=(\mathcal{U} \cup \mathcal{V}, \mathcal{E}^{-})$. Utilizing a design akin to [48], node representations are learned from positive graphs using a positive GNN, and from negative graphs using a negative GNN. The parameters are shared in the same perspective for positive (negative) GNNs. GAT model [60] is used as the graph encoder and it computes as follows:


(2) \begin{align*} & \begin{aligned} h_{i,k}^{(l+1),\zeta} = \text{GAT}_{k}^\zeta \left(h_{i,k}^{(l),\zeta}, \mathbf{G}_{k}^\zeta\right) \end{aligned} \end{align*}

(3) \begin{align*} & \begin{aligned} z_{i,k}^{\zeta} = \left[ h_{i,k}^{(0),\zeta} \parallel h_{i,k}^{(1),\zeta} \parallel \cdots \parallel h_{i,k}^{(L)} \right] \mathcal{W}_{k}^{\zeta} \end{aligned} \end{align*}

where $\zeta \in \left \{ +,- \right \}$, $k$ represents the $k$th augmented graph, $L$ denotes the number of GNN layers and $\mathcal{W}_{k}^{\zeta }$ is a learnable transformation matrix. $h_{i,k}^{(0),\zeta }$ denotes the input feature vector of the $i$th node and $h_{i,k}^{(l),\zeta }$ is the representation of the $i$th node for the $l$th layer. $z_{i,k}^{\zeta }$ represents the final representation of $i$th node in the $k$th augmented graph. Note that we used two GAT layers here.

### Objective loss

#### Contrastive loss

**Inter-view Contrastive Learning.** As mentioned earlier, we obtained four augmented graphs and further divided each augmented graph into positive and negative graphs for separate encoding. Since the positive and negative graphs contain different semantic properties, we define inter-view contrastive losses for both the positive and negative augmented graphs. In the following, we discuss the positive graphs in detail and define the losses for the negative graphs in a similar manner.

For any positive graph, in order to obtain more robust representations, CSGDN maximizes the agreements of the representations between the same node across different positive graphs while minimizing the representation similarities between different nodes. For example, the representation of the $i$th node in graph $ \mathbf{G}_{1}^{+}$ of the inter-set perspective, i.e. $z_{i, 1}^{+}$, should be consistent with representations generated from the same node in the other positive augmented graph $ \mathbf{G}_{2}^{+}$ in the same perspective, i.e. $z_{i, 2}^{+}$. Therefore, we treat the representations of the same node from other positive graphs within the same perspective as positive samples. Also, we want the representation of a node to be distinct from those of different nodes so we consider the representations of different nodes from other positive graphs within the same perspective as negative samples. Given a mini-batch $\mathcal{B}$ containing N nodes, the inter-view contrastive loss for positive augmented graphs is defined as follows, inspired by the InfoNCE loss [48, 61]:


(4) \begin{align*}& \begin{aligned} \mathcal{L}_{\text{inter}}^{+}=-\frac{1}{N} \sum_{i=1}^{N} \log \frac{\exp \left(\frac{\operatorname{sim}\left(z_{i, k}^{+}, z_{i, k^{\prime}}^{+}\right)}{\tau}\right)}{\sum_{j=1, j \neq i}^{N} \exp \left(\frac{\operatorname{sim}\left(z_{i, k}^{+}, z_{j, k^{\prime}}^{+}\right)}{\tau}\right)} \end{aligned}\end{align*}

where $z_{i, k}^{+}$ represents the representation of node $i$ in the $k$th augmented positive graph, sim($\cdot ,\cdot $) represents the similarity function between the two representations and $\tau $ denotes the preset temperature parameter.

Similarly, for the representation of the $i$th node in the graph $ \mathbf{G}_{1}^{-} $ of the inter-set perspective $z_{i,1}^{-}$, its inter-view positive samples are the representations generated from the same node from other negative graphs, and its inter-view negative samples are the ones generated from different nodes in other negative graphs. As the same, the inter-view contrastive loss for negative augmented graphs is defined as follows:


(5) \begin{align*}& \begin{aligned} \mathcal{L}_{\text{inter}}^{-} = -\frac{1}{N} \sum_{i=1}^{N} \log \frac{\exp \left( \frac{\operatorname{sim}\left(z_{i, k}^{-}, z_{i, k^{\prime}}^{-}\right)}{\tau} \right)}{\sum_{j=1, j \neq i}^{N} \exp \left( \frac{\operatorname{sim}\left(z_{i, k}^{-}, z_{j, k^{\prime}}^{-}\right)}{\tau} \right)} \end{aligned}\end{align*}

Combining the above two losses, we obtain the perspective specific contrastive loss:


(6) \begin{align*}& \begin{aligned} \mathcal{L}_{\text{inter}}=\mathcal{L}_{\text{inter}}^{+} + \mathcal{L}_{\text{inter}}^{-} \end{aligned}\end{align*}

**Intra-view contrastive learning.** In addition to maximizing the consistency of representations for the same node across different positive graphs or different negative graphs, we also design intra-view contrastive losses to allow the ultimate representation of each node to be close to the representations of the same node in positive graphs and far from the representations of the same node in negative graphs.

For node $v_{i}$, we generate the representation by concatenating all representations containing different information of diverse views, which is formulated as follows:


(7) \begin{align*}& \begin{aligned} z_{i} = g \left(z_{i,1}^{+} \parallel z_{i,2}^{+} \parallel z_{i,3}^{+} \parallel z_{i,4}^{+} \parallel z_{i,1}^{-} \parallel z_{i,2}^{-} \parallel z_{i,3}^{-} \parallel z_{i,4}^{-} \right), \end{aligned}\end{align*}

where $g$ is a two-layer MLP, and $z_{i} \in \mathbb{R}_{\text{d}}$ represents the final representation of node $v_{i}$.

To be specific, we treat the representations of the same node from other positive graphs as positive samples, while the representations of the same node from other negative graphs as negative samples. Given a mini-batch $\mathcal{B}$ containing N nodes, the intra-view contrastive objective is formally defined as follows:


(8) \begin{align*}& \begin{aligned} \mathcal{L}_{\text{intra}} = -\frac{1}{N} \sum_{i=1}^{N} \log \left( \frac{\sum_{m=1}^{M} \exp\left( \frac{\text{sim}(z_{i}, z_{i,m}^{+})}{\tau} \right)}{\sum_{m=1}^{M} \exp\left( \frac{\text{sim}(z_{i}, z_{i,m}^{-})}{\tau} \right)} \right), \end{aligned}\end{align*}

where $M$ denotes the number of graph views, which equals to 4 in this paper.

**Contrastive loss.** we generates the combined contrastive learning objective from the inter-view and intra-view contrastive learning objectives, and it is formulated as follows:


(9) \begin{align*}& \begin{aligned} \mathcal{L}_{\text{CL}} = (1-\alpha)\mathcal{L}_{\text{inter}} + \alpha\mathcal{L}_{\text{intra}}, \end{aligned}\end{align*}

where $\alpha $ is the weight coefficient that controls the significance between two losses.

#### Label loss

After obtaining the final node representations using Equation 9, we utilize a two-layer MLP to compute the sign scores between nodes from different sets:


(10) \begin{align*}& \begin{aligned} \hat{y} = \text{sigmoid}(\text{MLP}(z_{u_{i}} \parallel z_{v_{j}})), \end{aligned}\end{align*}

where $\hat{y}$ represents the predicted sign score of the link between nodes $u_{i} \in U$ and $v_{j} \in V$. The dimension of $\hat{y}$ is 3, representing the probabilities of predicting the edge sign as positive, negative, or neutral, respectively.

Following existing methodologies, the cross-entropy loss function is used for multi-class classification in link sign prediction:


(11) \begin{align*}& \begin{aligned} \mathcal{L_{\text{label}}}=-\frac{1}{N} \sum_{i=1}^{N} \sum_{c=1}^{C} y_{i, c} \log \left(\hat{y}_{i, c}\right) \end{aligned}\end{align*}

where c represents the class and $y_{\text{i,c}}$ is the ground truth label. The label c is defined as follows:

-1 indicates a negative association (negative regulation).
- 1 indicates a positive association (positive regulation).
- 0 indicates an undefined association which is not from the TWAS analysis process.

Note that the ground truth label are converted into one-hot encoding, with a value of 1 in the corresponding category position and 0 elsewhere.

#### Total loss

Finally, our model is trained using a joint loss function that integrates the link sign prediction loss and the contrastive learning loss:


(12) \begin{align*}& \begin{aligned} \mathcal{L} = \mathcal{L}_{\text{label}} + \beta \mathcal{L}_{\text{CL}}, \end{aligned}\end{align*}

where $\beta $ is a weight parameter that controls the relative importance of contrastive loss.




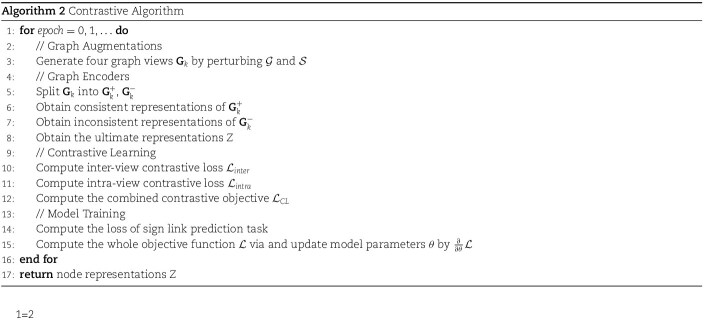



### MLP for genes without TWAS associations

**Figure 3:**
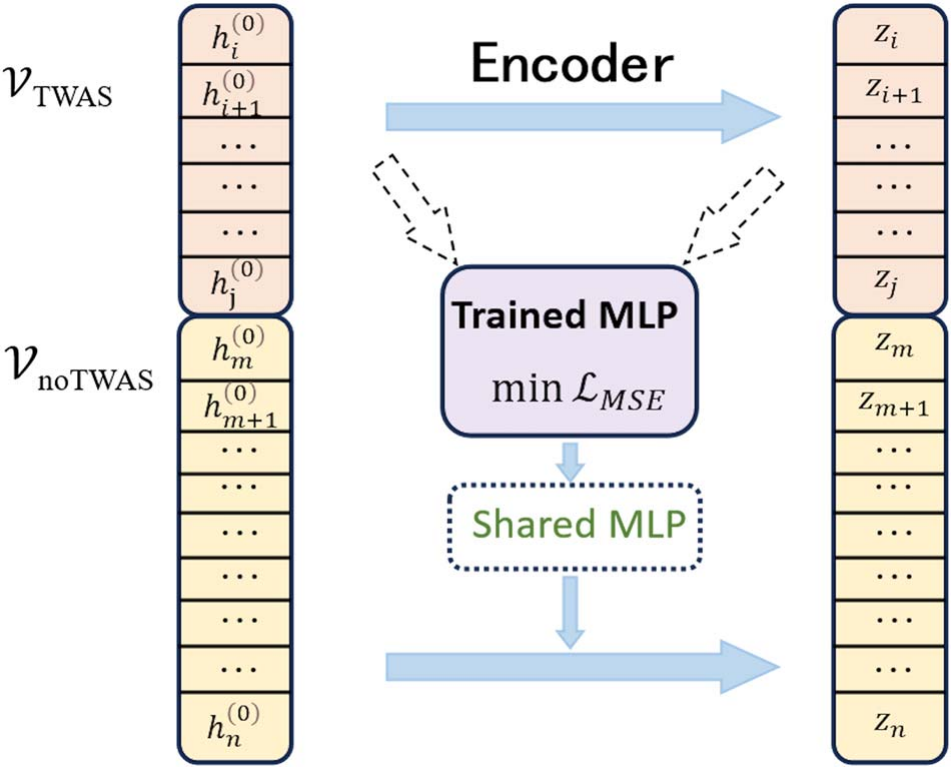
The frame for genes that can not be associated with phenotypes. For genes without TWAS associations, we train an MLP to map them into a shared space with TWAS-associated genes, which is trained by minimizing the MSE loss. The MLP is used to obtain the representation for genes lacking TWAS associations for gene–phenotype association predictions.

For those genes that lack TWAS associations, due to the lack of supervision from TWAS association information, the encoder trained specifically for genes in TWAS associations cannot be directly used for their encoding. To address this issue, we propose to train a multi-layer perceptron (MLP) to transform these genes that lack TWAS associations into a shared space with TWAS-associated genes:


(13) \begin{align*}& \mathcal{L}_{\text{MSE}} = \frac{1}{|\mathcal{V}_{\text{TWAS}}|} \sum_{v_{i} \in \mathcal{V}_{\text{TWAS}}} \left\| \text{MLP}(h_{i}^{(0)}) - z_{i} \right\|^{2}\end{align*}

where $h_{i}^{(0)}$ represents the input feature of gene $v_{i}$ in TWAS associations, and $z_{i}$ represents the final representation of gene $v_{i}$ after being encoded by the aforementioned TWAS framework. By minimizing the above Mean Squared Error (MSE) loss, we can learn the encoding capability of the main framework for TWAS-associated genes through MLP, which can be used for the encoding of genes lacking TWAS associations. Then we can obtain the representation of genes lacking TWAS associations through this MLP:


(14) \begin{align*}& z_{i} = \text{MLP}(h_{i}^{(0)})\end{align*}

where $v_{i} \in \mathcal{V}_{\text{noTWAS}}$. The specific process is shown in Fig. 3.

## Experiments

In this section, we present experiments on real-world datasets to evaluate the effectiveness of CSGDN in link sign prediction. We also compare its performance with the leading methods in both unsigned and signed graph neural networks. Specifically, our aim is to address the following questions:

- **Q1**: Does CSGDN outperform the advanced baselines?
- **Q2**: How does CSGDN perform with a small sample size and random noise?
- **Q3**: How do different model components affect the performance of CSGDN?
- **Q4**: Is CSGDN sensitive to hyperparameters?

### Experiment settings

**Baselines.** To validate the effectiveness of CSGDN, we compare our proposed model with several common methods in the fields of unsigned and signed graph neural networks.

- **Unsigned GNNs.** GCN [44] is a notable pioneering GNN model tailored for unsigned graphs, featuring an effective layer-wise propagation mechanism. GAT [60] utilizes masked self-attentional layers, allowing nodes to attend to their neighbors’ features with varying weights without costly matrix operations or prior knowledge of the graph structure. GRACE [62] proposes a novel unsupervised graph representation learning framework that leverages contrastive objectives at the node level, creating two graph views through corruption, and maximizing the agreement of node representations in these views, utilizing diverse contexts via a hybrid scheme on structure and attribute levels, and demonstrating superior performance over state-of-the-art methods.
- **Signed GNNs.** SGCN [39] utilizes balance theory to correctly aggregate and propagate the information across layers of a signed GCN model and generalizes GCN to signed graphs. SGCL [48] introduces new graph contrastive representation learning techniques tailored for signed graphs, utilizing balance theory and dual contrastive strategies to achieve superior node representations across diverse datasets, including social and online gaming networks. SGNNMD [34] utilizes signed graph neural networks to predict deregulation types of miRNA-disease associations, achieving competitive performance by integrating structural and biological features from a signed bipartite network.
- **Gene-phenotype association methods.** XGDAG [63] is an explainable neural network for gene-disease associations that utilizes positive unlabeled learning, integrates explainability with techniques to generate interpretable results, and shows robust performance on a protein-protein interaction network compared to traditional models. ModulePred [64] is also a deep learning method that combines graph augmentation, functional modules, and advanced GNN architectures to predict disease–gene associations.

**Hyperparameter settings.** we analyze the sensitivity of CSGDN to six key hyperparameters: $\alpha $, $\beta $, $feature$, $mask$, $predictor$, and $\tau $. The default configuration for each hyperparameter in the *G. hirsutum* dataset is as follows: $\alpha = 0.8$, $\beta = 0.01$, $feature = 64$, $mask = 0.4$, $predictor = \text{2-layer MLP}$, and $\tau = 0.05$. The settings for the *B. napus* dataset are $\alpha = 0.2$, $\beta = 0.1$, $feature = 32$, $mask = 0.8$, $predictor = \text{1-layer MLP}$, and $\tau = 0.05$. For the *T. turgidum* dataset, the hyperparameter settings are identical to those used for *G. hirsutum*. These settings were determined based on the model’s overall highest AUC score. The AUC value is used as the primary metric to assess the sensitivity of the model to changes in these hyperparameters.

**Reducing sample size.** We randomly extract 80% edges of the *G. hirsutum* dataset. CSGDN shows excellent results to face with small sample size in the link sign prediction task. This setting suggests that CSGDN is effective in situations with limited data availability, demonstrating its robustness when working with smaller datasets. The result is shown in Table 7.

**Random noise.** To demonstrate that our model CSGDN has an outstanding performance when resisting interference, we achieve the effect of random perturbation (Ptb) to simulate noise by randomly flipping a certain proportion of edge signs in the *G. hirsutum* dataset. In this experiment, we set the proportion as 10% and 20%. This setting to illustrate the performance of CSGDN is particularly important. The robustness of CSGDN ensures that it can still provide reliable predictions in such challenging conditions. The result is shown in Table 8.

**Task and evaluation metrics.** We use AUC, F1, Micro, Macro, AUPR, and Precision to evaluate the results on the link sign prediction task. For each dataset, we randomly split edges into a training set and a testing set with a 8:2 ratio. Note that superior performance is indicated by higher values for all four evaluation metrics.

### Experiment results

**Performance of CSGDN compared to baselines (Q1).** To answer **Q1**, we compare CSGDN with current state-of-the-art methods. We primarily use two types of GNN frameworks as baselines: unsigned GNN and signed GNN. For unsigned GNNs, we used GCN, GAT, and GRACE, while for signed GNNs, we employed SGCN, SGCL, and SGNNMD. Also we shows the results of gene–phenotype association prediction methods, XGDAG and ModulePred. We use link prediction as the evaluation task, with AUC, F1, Micro, Macro, AUPR, and Precision as the evaluation metrics for model performance. Across three common crop datasets (*G. hirsutum*, *B. napus*, and *T. turgidum*), the evaluation metrics of CSGDN generally outperform those of the state-of-the-art baselines. As shown in Table 2, CSGDN demonstrates strong performance in the link prediction task in crop datasets. And as shown in Table 3, we compare our CSGDN with two deep-learning methods, which also demonstrates a better performance.

**Table 2:** Link sign prediction results (average ${\pm }$ standard deviation) with AUC, F1, Micro, Macro, AUPR, and Precision (%) on three datasets from previous studies

**Datasets**	**Metrics**	**Unsigned GNNs**			**Signed GNNs**			**Our model**
		**GCN**	**GAT**	**GRACE**	**SGCN**	**SGCL**	**SGNNMD**	**CSGDN**
*G. hirsutum*	AUC	0.6764 ${\pm }$ 0.0225	0.6649 ${\pm }$ 0.0169	0.6829 ${\pm }$ 0.0056	0.6882 ${\pm }$ 0.0135	0.6905 ${\pm }$ 0.0164	0.6802 ${\pm }$ 0.1359	**0.7764 ${\pm }$ 0.0047**
	F1	0.5646 ${\pm }$ 0.0542	0.5166 ${\pm }$ 0.0387	0.5529 ${\pm }$ 0.0157	0.5891 ${\pm }$ 0.0262	0.5806 ${\pm }$ 0.0275	**0.8337 ${\pm }$ 0.0346**	0.7411 ${\pm }$ 0.0152
	Micro	0.7158 ${\pm }$ 0.0146	0.7203 ${\pm }$ 0.0129	0.7353 ${\pm }$ 0.0030	0.7263 ${\pm }$ 0.0113	0.7353 ${\pm }$ 0.0146	**0.7874 ${\pm }$ 0.0783**	0.7748 ${\pm }$ 0.0160
	Macro	0.6755 ${\pm }$ 0.0230	0.6598 ${\pm }$ 0.0220	0.6824 ${\pm }$ 0.0078	0.6918 ${\pm }$ 0.0147	0.6936 ${\pm }$ 0.0185	0.6168 ${\pm }$ 0.0858	**0.7696 ${\pm }$ 0.0160**
	AUPR	0.7252 ${\pm }$ 0.0322	0.7461 ${\pm }$ 0.0190	0.7635 ${\pm }$ 0.0075	0.7256 ${\pm }$ 0.0158	0.7438 ${\pm }$ 0.0193	0.7166 ${\pm }$ 0.1147	**0.7928 ${\pm }$ 0.0110**
	Precision	0.7673 ${\pm }$ 0.1018	0.8663 ${\pm }$ 0.0408	**0.8812 ${\pm }$ 0.0265**	0.7570 ${\pm }$ 0.0399	0.8132 ${\pm }$ 0.0261	0.8240 ${\pm }$ 0.0538	0.7108 ${\pm }$ 0.0637
*B. napus*	AUC	0.5000 ${\pm }$ 0.0000	0.5000 ${\pm }$ 0.0000	0.5000 ${\pm }$ 0.0000	0.6212 ${\pm }$ 0.0000	0.5000 ${\pm }$ 0.0000	0.4341 ${\pm }$ 0.1168	**0.6610 ${\pm }$ 0.0191**
	F1	**0.9130 ${\pm }$ 0.0000**	**0.9130 ${\pm }$ 0.0000**	0.8773 ${\pm }$ 0.0000	0.9043 ${\pm }$ 0.0000	**0.9130 ${\pm }$ 0.0000**	0.0323 ${\pm }$ 0.0438	0.8042 ${\pm }$ 0.0765
	Micro	**0.8400 ${\pm }$ 0.0000**	**0.8400 ${\pm }$ 0.0000**	0.7815 ${\pm }$ 0.0000	0.8344 ${\pm }$ 0.0000	**0.8400 ${\pm }$ 0.0000**	0.6676 ${\pm }$ 0.2647	0.7192 ${\pm }$ 0.0838
	Macro	0.4565 ${\pm }$ 0.0000	0.4565 ${\pm }$ 0.0000	0.4387 ${\pm }$ 0.0000	**0.6472 ${\pm }$ 0.0000**	0.4565 ${\pm }$ 0.0000	0.3882 ${\pm }$ 0.1246	0.6368 ${\pm }$ 0.0520
	AUPR	**0.9200 ${\pm }$ 0.0000**	**0.9200 ${\pm }$ 0.0000**	0.8972 ${\pm }$ 0.0000	0.9126 ${\pm }$ 0.0000	**0.9200 ${\pm }$ 0.0000**	0.3340 ${\pm }$ 0.2158	0.9054 ${\pm }$ 0.0109
	Precision	0.8400 ${\pm }$ 0.0000	0.8400 ${\pm }$ 0.0000	0.7815 ${\pm }$ 0.0000	0.8252 ${\pm }$ 0.0000	0.8400 ${\pm }$ 0.0000	0.0317 ${\pm }$ 0.0396	**0.8622 ${\pm }$ 0.0121**
*T. turgidum*	AUC	0.5000 ${\pm }$ 0.0000	0.5000 ${\pm }$ 0.0000	0.5000 ${\pm }$ 0.0000	0.4867 ${\pm }$ 0.0286	0.5000 ${\pm }$ 0.0000	0.6000 ${\pm }$ 0.1265	**0.6263 ${\pm }$ 0.0948**
	F1	0.8235 ${\pm }$ 0.0000	0.8235 ${\pm }$ 0.0000	**0.8636 ${\pm }$ 0.0000**	0.8088 ${\pm }$ 0.0294	0.8235 ${\pm }$ 0.0000	0.2643 ${\pm }$ 0.3286	0.8337 ${\pm }$ 0.0346
	Micro	0.7000 ${\pm }$ 0.0000	0.7000 ${\pm }$ 0.0000	0.7600 ${\pm }$ 0.0000	0.6800 ${\pm }$ 0.0400	0.7000 ${\pm }$ 0.0000	**0.8431 ${\pm }$ 0.0512**	0.7440 ${\pm }$ 0.0456
	Macro	0.4118 ${\pm }$ 0.0000	0.4118 ${\pm }$ 0.0000	0.4318 ${\pm }$ 0.0000	0.4044 ${\pm }$ 0.0147	0.4118 ${\pm }$ 0.0000	0.5881 ${\pm }$ 0.1778	**0.6168 ${\pm }$ 0.0858**
	AUPR	0.8500 ${\pm }$ 0.0000	0.8500 ${\pm }$ 0.0000	0.8800 ${\pm }$ 0.0000	0.8424 ${\pm }$ 0.0152	0.8500 ${\pm }$ 0.0000	0.6785 ${\pm }$ 0.1010	**0.8943 ${\pm }$ 0.0212**
	Precision	0.7000 ${\pm }$ 0.0000	0.7000 ${\pm }$ 0.0000	0.7600 ${\pm }$ 0.0000	0.6933 ${\pm }$ 0.0133	0.7000 ${\pm }$ 0.0000	0.4000 ${\pm }$ 0.4899	**0.8240 ${\pm }$ 0.0539**

**Table 3:** Link sign prediction results (average ${\pm }$ standard deviation) with AUC, F1, Micro, Macro, AUPR, and Precision (%) on three datasets from previous studies, comparing with two deep-learning methods in the field of gene–phenotype association

**Datasets**	**Metrics**	**Gene-phenotype association methods**		**Our model**
		**XGDAG**	**ModulePred**	**CSGDN**
*G. hirsutum*	AUC	0.6327 ${\pm }$ 0.0775	0.7329 ${\pm }$ 0.0358	**0.7764 ${\pm }$ 0.0047**
	F1	0.5102 ${\pm }$ 0.1037	0.6491 ${\pm }$ 0.0767	**0.7411 ${\pm }$ 0.0152**
	Micro	0.6632 ${\pm }$ 0.1324	0.7579 ${\pm }$ 0.0074	**0.7748 ${\pm }$ 0.0160**
	Macro	0.5803 ${\pm }$ 0.1567	0.7267 ${\pm }$ 0.0240	**0.7696 ${\pm }$ 0.0160**
	AUPR	0.7748 ${\pm }$ 0.0402	**0.7988 ${\pm }$ 0.0260**	0.7928 ${\pm }$ 0.0110
	Precision	**0.8983 ${\pm }$ 0.0508**	0.8908 ${\pm }$ 0.0612	0.8622 ${\pm }$ 0.0121
*B. napus*	AUC	**0.6707 ${\pm }$ 0.0769**	0.6570 ${\pm }$ 0.0863	0.6610 ${\pm }$ 0.0191
	F1	0.7114 ${\pm }$ 0.3229	0.7240 ${\pm }$ 0.3137	**0.8042 ${\pm }$ 0.0765**
	Micro	0.6901 ${\pm }$ 0.2225	0.6993 ${\pm }$ 0.2207	**0.7192 ${\pm }$ 0.0838**
	Macro	0.6106 ${\pm }$ 0.1972	0.5990 ${\pm }$ 0.1894	**0.6368 ${\pm }$ 0.0520**
	AUPR	**0.9169 ${\pm }$ 0.0112**	0.9161 ${\pm }$ 0.0135	0.9054 ${\pm }$ 0.0109
	Precision	**0.8983 ${\pm }$ 0.0508**	0.8908 ${\pm }$ 0.0612	0.8622 ${\pm }$ 0.0121
*T. turgidum*	AUC	0.5096 ${\pm }$ 0.0193	0.5000 ${\pm }$ 0.0000	**0.6263 ${\pm }$ 0.0948**
	F1	0.5982 ${\pm }$ 0.3489	0.6909 ${\pm }$ 0.3455	**0.8337 ${\pm }$ 0.0346**
	Micro	0.5840 ${\pm }$ 0.2214	0.6560 ${\pm }$ 0.2080	**0.7440 ${\pm }$ 0.0456**
	Macro	0.3778 ${\pm }$ 0.0929	0.3842 ${\pm }$ 0.0953	**0.6168 ${\pm }$ 0.0858**
	AUPR	0.6227 ${\pm }$ 0.3126	0.6080 ${\pm }$ 0.3040	**0.8240 ${\pm }$ 0.0539**
	Precision	0.8696 ${\pm }$ 0.0207	0.8800 ${\pm }$ 0.0000	**0.8943 ${\pm }$ 0.0212**

**Figure 4:**
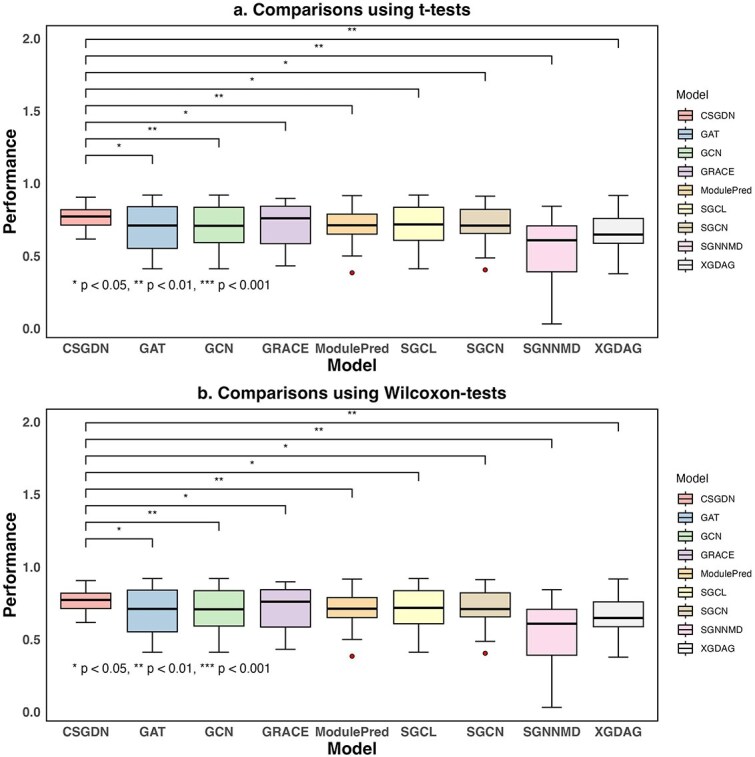
Comparisons between CSGDN and eight baseline models using two statistical tests. The asterisk (’$\ast $’) indicates the significance levels observed in the different tests. Figure 4(a) shows the results of t-tests, while Fig. 4(b) presents the results of Wilcoxon tests.

What’s more, we conduct both t-tests and Wilcoxon signed-rank tests to analyze these results. Specifically, we employ the t.test() function to perform paired two-sample t-tests, which evaluates whether there are statistically significant differences. Additionally, we use the wilcox.test() function to conduct the Wilcoxon signed-rank tests, to assess whether the differences between paired observations are significantly different from zero. Notably, the p-values obtained from these analyses are all less than 0.05, indicating that the observed differences are statistically significant. This strong evidence supports the conclusion that our proposed CSGDN model outperforms most of the baseline methods, showing its superior performance. The results of these statistical analyses are presented in Table 4 and Fig. 4.

**Table 4:** P-value results of comparisons between CSGDN and 8 baseline models using two statistical tests

	**GAT**	**GCN**	**GRACE**	**ModulePred**	**SGCL**	**SGCN**	**SGNNMD**	**XGDAG**
t-test	0.0176	0.00688	0.0226	0.00789	0.0151	0.0143	0.00273	0.00158
wilcox-test	0.0134	0.00797	0.0216	0.00385	0.0134	0.0152	0.00280	0.00200

Furthermore, we perform CSGDN at different five fiber developmental timepoints in *G. hirsutum*, including 4 DPA, 8 DPA, 12 DPA, 16 DPA, and 20 DPA, as shown in Table 5. For the *G. hirsutum* dataset, we further validate our predictions by comparing them with the results obtained using the COLOC and SMR methodologies. It is important to note that the datasets used for COLOC and SMR, as well as the dataset used for our model, are derived from the same study [11]. Notably, the overlapping genes between our model’s predictions and those identified by the COLOC and SMR methods achieved a 100% match, further substantiating the reliability and accuracy of our approach. As shown in Table 6, the COLOC method identified 19 different associations between genes and phenotypes, and the SMR method identified 9 associations, all of which are overlapped entirely with the results identified by our new model.

**Table 5:** CSGDN’s performance (average ${\pm }$ standard deviation) at five fiber developmental timepoints in *G. hirsutum* containing 4 DPA, 8 DPA, 12 DPA, 16 DPA, 20 DPA

	**4 DPA**	**8 DPA**	**12 DPA**	**16 DPA**	**20 DPA**
AUC	0.7764 ${\pm }$ 0.0047	**0.7888 ${\pm }$ 0.0126**	0.7598 ${\pm }$ 0.0501	0.7829 ${\pm }$ 0.0155	0.7770 ${\pm }$ 0.0310
F1	0.7411 ${\pm }$ 0.0152	0.7641 ${\pm }$ 0.0189	0.7539 ${\pm }$ 0.0530	**0.7858 ${\pm }$ 0.0290**	0.7658 ${\pm }$ 0.0265
Micro	0.7748 ${\pm }$ 0.0160	**0.7856 ${\pm }$ 0.0130**	0.7571 ${\pm }$ 0.0501	0.7702 ${\pm }$ 0.0198	0.7726 ${\pm }$ 0.0299
Macro	0.7686 ${\pm }$ 0.0110	**0.7856 ${\pm }$ 0.0145**	0.7571 ${\pm }$ 0.0560	0.7702 ${\pm }$ 0.0222	0.7726 ${\pm }$ 0.0334
AUPR	0.7928 ${\pm }$ 0.0098	0.8571 ${\pm }$ 0.0149	0.8114 ${\pm }$ 0.0359	**0.8778 ${\pm }$ 0.0096**	0.8619 ${\pm }$ 0.0253
Precision	0.7108 ${\pm }$ 0.0637	**0.8710 ${\pm }$ 0.0457**	0.7363 ${\pm }$ 0.0605	0.8717 ${\pm }$ 0.0296	0.8433 ${\pm }$ 0.0636

**Table 6:** Comparative results with the COLOC method and SMR method in the *G. hirsutum* dataset

**Phenotype**	**Timepoint**	**Gene ID**	**COLOC.PPH4**	**CSGDN results**	**Negative probability**	**None-association probability**	**Positive Probability**
FL	8 DPA	Ghir_A11G010270	0.845	up	0.0264	0.1966	0.7770
FL	12 DPA	Ghir_A11G010270	0.871	up	0.0004	0.0132	0.9865
FL	16 DPA	Ghir_A11G010320	0.832	down	0.9460	0.0515	0.0025
FL	12 DPA	Ghir_D05G007220	0.925	up	0.0004	0.0137	0.9858
FL	20 DPA	Ghir_D05G007220	0.932	up	0.0040	0.1698	0.8262
FS	8 DPA	Ghir_A11G010270	0.820	up	0.0243	0.1599	0.8158
FS	12 DPA	Ghir_A11G010270	0.850	up	0.0004	0.0133	0.9863
FS	20 DPA	Ghir_A11G010270	0.829	up	0.0097	0.2857	0.7046
FS	16 DPA	Ghir_A11G010320	0.817	down	0.9478	0.0497	0.0025
FS	4 DPA	Ghir_A11G010750	0.874	up	0.0009	0.1874	0.8117
FE	12 DPA	Ghir_A01G017200	0.960	up	0.0006	0.0172	0.9822
FE	12 DPA	Ghir_A01G017360	0.887	down	0.9919	0.0067	0.0014
FE	16 DPA	Ghir_A01G017360	0.864	down	0.8944	0.0973	0.0084
FE	20 DPA	Ghir_A01G017360	0.827	down	0.8430	0.1539	0.0031
FE	4 DPA	Ghir_A01G017420	0.802	down	0.8279	0.1718	0.0003
FE	16 DPA	Ghir_D05G022050	0.965	up	0.0003	0.0541	0.9456
FE	20 DPA	Ghir_D05G022050	0.970	up	0.0061	0.3523	0.6417
FE	8 DPA	Ghir_D07G001340	0.839	down	0.9731	0.0253	0.0016
FU	20 DPA	Ghir_D12G019870	0.862	up	0.0099	0.2343	0.7558
**Phenotype**	**Timepoint**	**Gene ID**	**SMR.p-value**	**CSGDN results**	**Negative probability**	**None-association probability**	**Positive Probability**
FL	12 DPA	Ghir_D05G007220	2.36E-7	up	0.0004	0.0137	0.9858
FL	20 DPA	Ghir_D05G007220	8.84E-8	up	0.0040	0.1698	0.8262
FS	12 DPA	Ghir_A01G017200	1.51E-7	up	0.0006	0.0173	0.9821
FS	12 DPA	Ghir_A11G010270	3.41E-10	up	0.0004	0.0133	0.9863
FS	20 DPA	Ghir_A11G010270	2.01E-9	up	0.0097	0.2857	0.7046
FS	4 DPA	Ghir_A11G010750	3.41E-10	up	0.0009	0.1874	0.8117
FE	12 DPA	Ghir_A01G017200	2.89E-6	up	0.0006	0.0172	0.9822
FE	12 DPA	Ghir_A01G017360	4.46E-6	down	0.9919	0.0067	0.0014
FE	20 DPA	Ghir_D05G022050	4.77E-6	up	0.0061	0.3523	0.6417

**Performance of CSGDN when addressing small sample size and random noise (Q2).** As shown in Tables 7 and 8, we address **Q2** by verifying that CSGDN can effectively overcome two issues: the costs of samples and noise. For the first issues, we randomly reduce the sample size of the *G. hirsutum* dataset to 80% and subsequently divide this dataset into training and testing sets. The results presented in Table 7 indicate that CSGDN outperforms all baselines on *G. hirsutum* datasets with randomly reduced sample sizes, demonstrating its effectiveness in handling small sample datasets. This suggests that we can reduce the experimental costs and durations by minimizing the sample size and still achieving excellent prediction outcomes. Then for noise, we utilize two *G. hirsutum* datasets with the proportion of 10% and 20% perturbations. As shown in Table 8, our model CSGDN outperforms most of the baselines. This reflects that our model has strong anti-interference ability against various types of noise through contrastive learning methods.

**Table 7:** Link sign prediction results (average ${\pm }$ standard deviation) with AUC, F1, Micro, and Macro (%) for randomly sampled 80% *G. hirsutum* dataset

	**AUC**	**F1**	**Micro**	**Macro**
GCN	0.5883 ${\pm }$ 0.0182	0.4084 ${\pm }$ 0.0932	0.6562 ${\pm }$ 0.0393	0.5792 ${\pm }$ 0.0317
GAT	0.5549 ${\pm }$ 0.0244	0.3042 ${\pm }$ 0.1045	0.6315 ${\pm }$ 0.1417	0.4811 ${\pm }$ 0.1058
GRACE	0.6967 ${\pm }$ 0.0208	0.6203 ${\pm }$ 0.0330	0.7098 ${\pm }$ 0.0504	0.6849 ${\pm }$ 0.0378
SGCN	0.5832 ${\pm }$ 0.0478	0.4134 ${\pm }$ 0.0885	0.6472 ${\pm }$ 0.0208	0.5793 ${\pm }$ 0.0431
SGCL	0.6271 ${\pm }$ 0.0429	0.4980 ${\pm }$ 0.0975	0.6360 ${\pm }$ 0.0464	0.5926 ${\pm }$ 0.0174
SGNNMD	0.5988 ${\pm }$ 0.1210	0.5035 ${\pm }$ 0.2912	0.5964 ${\pm }$ 0.1242	0.5231 ${\pm }$ 0.1843
CSGDN	**0.7495 ${\pm }$ 0.0339**	**0.6884 ${\pm }$ 0.0407**	**0.7574 ${\pm }$ 0.0515**	**0.7406 ${\pm }$ 0.0420**

**Table 8:** Link sign prediction results (average ${\pm }$ standard deviation) with AUC, F1, Micro and Macro (%) for the *G. hirsutum* dataset with perturbations

	**Ptb(%)**	**Unsigned GNNs**			**Signed GNNs**			**Our model**
		**GCN**	**GAT**	**GRACE**	**SGCN**	**SGCL**	**SGNNMD**	**CSGDN**
AUC	10	0.6714 ${\pm }$ 0.0116	0.6649 ${\pm }$ 0.0169	0.6759 ${\pm }$ 0.0163	0.6994 ${\pm }$ 0.0138	0.6850 ${\pm }$ 0.0221	0.6258 ${\pm }$ 0.1102	**0.7066 ${\pm }$ 0.0300**
F1		0.5424 ${\pm }$ 0.0302	0.5166 ${\pm }$ 0.0387	0.5435 ${\pm }$ 0.0309	0.6046 ${\pm }$ 0.0330	0.5663 ${\pm }$ 0.0429	0.5547 ${\pm }$ 0.2151	**0.6498 ${\pm }$ 0.0562**
Micro		0.7203 ${\pm }$ 0.0129	0.7203 ${\pm }$ 0.0129	0.7278 ${\pm }$ 0.0138	**0.7368 ${\pm }$ 0.0106**	0.7323 ${\pm }$ 0.0169	0.6045 ${\pm }$ 0.1409	0.6987 ${\pm }$ 0.0253
Macro		0.6702 ${\pm }$ 0.0150	0.6598 ${\pm }$ 0.0220	0.6748 ${\pm }$ 0.0196	**0.7033 ${\pm }$ 0.0159**	0.6863 ${\pm }$ 0.0259	0.5891 ${\pm }$ 0.1427	0.6987 ${\pm }$ 0.0253
AUC	20	0.6030 ${\pm }$ 0.0454	0.6313 ${\pm }$ 0.0176	0.6515 ${\pm }$ 0.0061	0.6225 ${\pm }$ 0.0312	0.6518 ${\pm }$ 0.0198	0.6063 ${\pm }$ 0.1025	**0.7209 ${\pm }$ 0.0298**
F1		0.4939 ${\pm }$ 0.0901	0.4863 ${\pm }$ 0.0317	0.5320 ${\pm }$ 0.0047	0.5576 ${\pm }$ 0.0403	0.5840 ${\pm }$ 0.0338	0.6420 ${\pm }$ 0.1803	**0.6501 ${\pm }$ 0.0530**
Micro		0.6286 ${\pm }$ 0.0400	0.6797 ${\pm }$ 0.0162	0.6932 ${\pm }$ 0.0111	0.6316 ${\pm }$ 0.0293	0.6602 ${\pm }$ 0.0573	0.6180 ${\pm }$ 0.1165	**0.7444 ${\pm }$ 0.0190**
Macro		0.5968 ${\pm }$ 0.0468	0.6268 ${\pm }$ 0.0207	0.6517 ${\pm }$ 0.0053	0.6205 ${\pm }$ 0.0306	0.6364 ${\pm }$ 0.0408	0.5847 ${\pm }$ 0.1244	**0.7236 ${\pm }$ 0.0289**

### Ablation study

We conducted an ablation study to assess the effectiveness of different components in our proposed model to answer **Q3**. In this subsection, we employ the sign perturbation as the graph-augmentation method to analyze performance. Specifically, we compare CSGDN with its three variants: $\text{CSGDN}_{\mathrm{w/o\ diffuse}}$, $\text{CSGDN}_{\mathrm{w/o\ aug}}$, and $\text{CSGDN}_{\mathrm{w/o\mathcal{L}_{CL}}}$, which are defined as follows:

-
$\text{CSGDN}_{\mathrm{w/o\ diffuse}}$ : the graph diffusion in contrastive learning is removed, and instead we utilize the same two original graphs without the diffusion step for the next augmentation step.
-
$\text{CSGDN}_{\mathrm{w/o\ aug}}$ : the graph augmentation step is removed. In this variant, the graphs $\mathcal{G}$ and $\mathcal{S}$ instead of the augmented graphs $\mathbf{G_{K}}$ are exploited during training.
-
$\text{CSGDN}_{\mathrm{w/o\mathcal{L}_{CL}}}$ : the part of contrastive learning is removed and ignores contrastive loss.

As shown in Table 9, we demonstrate that all three components are essential for CSGDN’s performance. Each component plays a unique role in enhancing model’s ability.

**Table 9:** The AUC performances (average ${\pm }$ standard deviation) with CSGDN and its variants

	CSGDN	$\text{CSGDN}_{\mathrm{w/o\ diffuse}}$	$\text{CSGDN}_{\mathrm{w/o\ aug}}$	$\text{CSGDN}_{\mathrm{w/o\mathcal{L}_{CL}}}$
*G. hirsutum*	**0.7811 ${\pm }$ 0.0116**	0.7624 ${\pm }$ 0.0179	0.7712 ${\pm }$ 0.0551	0.7297 ${\pm }$ 0.0208
*B. napus*	0.6615 ${\pm }$ 0.0495	0.6485 ${\pm }$ 0.0400	**0.6700 ${\pm }$ 0.0206**	0.6204 ${\pm }$ 0.0017
*T. turgidum*	**0.5982 ${\pm }$ 0.0787**	0.5175 ${\pm }$ 0.0351	0.5333 ${\pm }$ 0.0887	0.4956 ${\pm }$ 0.0428
80% *G. hirsutum*	**0.7495 ${\pm }$ 0.0339**	0.6998 ${\pm }$ 0.0365	0.7355 ${\pm }$ 0.0184	0.6870 ${\pm }$ 0.0327

### Hyper-parameters analysis

To answer **Q4**, we analyze the sensitivity of CSGDN to six key hyperparameters: $\alpha $, $\beta $, $feature$, $mask$, $predictor$, and $\tau $.

**Figure 5:**
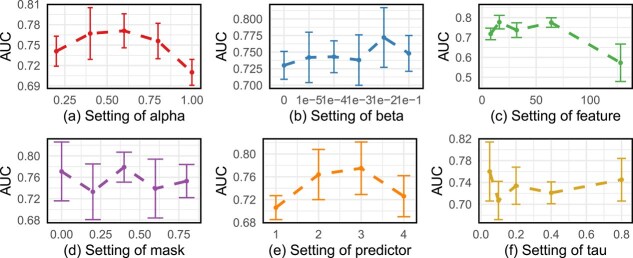
Hyperparameter sensitivity CSGDN in the *G. hirsutum* dataset.

The hyperparameter $\alpha $, shown in Fig. 5(a), serves as the weight coefficient that balances the significance of the inter-view contrastive loss and intra-view contrastive loss. We evaluate the performance of the model with $\alpha $ set to values from the set ${0.2, 0.4, 0.6, 0.8, 1.0}$. We find that the model performs better when $\alpha $ is between 0.5 and 0.8, while anything outside of this range decreases performance. Therefore, for this dataset, intra-view, i.e. learning a consistent representation across augmented graphs, is more important.

The hyperparameter $\beta $, shown in Fig. 5(b), controls the trade-off in the joint loss function between the link sign prediction loss ($\mathcal{L}_{label}$) and the contrastive learning loss ($\mathcal{L}_{CL}$). We vary $\beta $ over the set ${0, 0.1, 0.01, 0.001, 0.0001, 0.00001}$ and observe that the highest AUC score is achieved when $\beta = 0.01$. We find that the model performance tends to increase when $\beta> 0.01$, and the model reaches its best performance at $\beta = 0.01$, followed by a decrease in performance. This illustrates the importance of the contrast learning component in our model CSGDN, which decreases when the contrast learning effect on model performance is extreme, i.e. when the $\beta $ value is too small or too large.

The hyperparameter $feature$, shown in Fig. 5(c), refers to the node embedding dimension. We test six different node embedding dimensions, ranging from 8 to 128, and evaluate their impact on model performance. We find that the model performance decreases extremely when $feature = 128$, which may be due to feature sparsity caused by the elevated dimension of the feature space, which prevents the model from effectively establishing correlations between nodes.

The hyperparameter $mask$, shown in Fig. 5(d), represents the ratio of edges randomly dropped from both the original and diffusion graphs. We explore the values of $mask$ in the set ${0, 0.2, 0.4, 0.6, 0.8}$ and find that the model performs optimally when the mask ratio is set to 0.4. We find that the model performance fluctuates as the mask ratio increases, but generally performs better when the mask ratio is small. When the mask ratio is large, too much information is lost, resulting in incomplete information and lower model performance.

The hyperparameter $predictor$, shown in Fig. 5(e), determines the best architecture of the downstream link prediction task, allowing for variations among a one-layer MLP, two-layer MLP, three-layer MLP, and four-layer MLP. The number of layers directly influences the prediction accuracy of the model. We found that as the number of layers of $predictor$ increases, the performance of the model first increases and then decreases, and reaches the highest value in the three-layer MLP, while the four-layer MLP may be due to the overfitting of the model due to the excessive number of layers of the neural network, which ultimately manifests itself in the decrease in model performance on the test set.

Finally, the hyperparameter $\tau $, shown in Fig. 5(f), is the temperature parameter used in the contrastive loss function to regulate the similarity contrast between positive and negative edges. We tested $\tau $ values from the set ${0.05, 0.1, 0.2, 0.4, 0.8}$, and the default value of $\tau = 0.05$ produces the best performance. We find that the model performance is better when the $\tau $ value is lower and generally lower when $\tau> 0.05$, which also indicates that this dataset needs to learn a consistent representation as much as possible in comparative learning, and the smoothing operation here will reduce the performance.

### Case study

In this section, we start a case study focusing on *G. hirsutum* genes that associated with the four types of phenotypes, including the FE rate, the FU, the FS, and the FL. Using the optimal hyperparameter configurations described, we utilize the associations between *G. hirsutum* genes and the four types of phenotypes predicted by the TWAS process as the training set to train the CSGDN TWAS frame. Then, the randomly selected gene–phenotype associations types that TWAS cannot calculate in *G. hirsutum* are used as irrelevant associations. CSGDN can predict probabilities of three types of associations, including up-association, down-association, and none-association. As shown in Table 10, we randomly list eight associations for each phenotype. We can observe from Table 10 that the prediction results of our model contain three probability values, namely positive-association probability, negative-association probability, and none-association gene–phenotype probability, with the sum of these three equaling 1. We can simply determine which type of up-down-none association belongs to a certain gene and a certain phenotype by selecting the largest of the three probability values. In addition, a larger probability indicates stronger relevance between a specific gene–phenotype association. We take the result associations from the original TWAS results as a reference for our predictions. In other words, if the associations from the original TWAS testing datasets match those of the CSGDN predictions, it indicates that our model has made accurate predictions. Therefore, the case studies demonstrate the usefulness of CSGDN in discovering novel gene–phenotype associations and can be validated by TWAS results.

**Table 10:** Eight genes associated with *G. hirsutum* phenotypes and their types predicted by CSGDN

**Gene**	**Phenotype**	**TWAS**	**CSGDN**	** $P_{\text{down}}$ **	** $P_{\text{none}}$ **	** $P_{\text{up}}$ **
Ghir_A13G012290	FE	Up	Down	0.672	0.033	0.294
Ghir_D02G002560	FE	Up	Up	0.012	0.007	0.982
Ghir_A11G026810	FE	Up	Up	0.012	0.007	0.981
Ghir_A09G016050	FE	Up	Up	0.452	0.035	0.513
Ghir_A12G025670	FE	Up	Down	0.483	0.035	0.482
Ghir_A09G014910	FE	Down	Down	0.971	0.011	0.018
Ghir_A01G013950	FE	Down	Down	0.989	0.006	0.005
Ghir_D01G000640	FE	Down	Down	0.928	0.018	0.054
Ghir_A02G005400	FU	Down	Up	0.043	0.013	0.944
Ghir_A05G041310	FU	Up	Up	0.014	0.007	0.979
Ghir_A04G002650	FU	Up	Up	0.031	0.011	0.958
Ghir_D03G012470	FU	Up	Up	0.012	0.007	0.981
Ghir_A12G019480	FU	Down	Down	0.990	0.006	0.004
Ghir_A12G019760	FU	Down	Up	0.019	0.009	0.972
Ghir_A01G018190	FU	Down	Down	0.990	0.006	0.004
Ghir_A01G017780	FU	Down	Down	0.991	0.005	0.004
Ghir_D09G001870	FS	Up	Up	0.014	0.007	0.979
Ghir_D07G018070	FS	Up	Up	0.089	0.019	0.892
Ghir_D05G001070	FS	Up	Up	0.012	0.007	0.982
Ghir_D07G018070	FS	Down	Up	0.018	0.008	0.973
Ghir_D07G002590	FS	Down	Down	0.493	0.035	0.472
Ghir_D06G018900	FS	Down	Down	0.991	0.005	0.004
Ghir_D13G017280	FS	Down	Down	0.991	0.005	0.004
Ghir_A01G016960	FS	Down	Down	0.989	0.006	0.005
Ghir_A09G017330	FL	Up	Up	0.010	0.006	0.984
Ghir_D07G002590	FL	Up	Up	0.010	0.006	0.984
Ghir_D01G006880	FL	Down	Up	0.282	0.031	0.686
Ghir_D05G006550	FL	Down	Down	0.990	0.006	0.004
Ghir_A01G012860	FL	Down	Down	0.589	0.035	0.376
Ghir_A01G017490	FL	Down	Down	0.981	0.009	0.011
Ghir_A01G013320	FL	Down	Down	0.987	0.007	0.006
Ghir_A11G001240	FL	Down	Up	0.038	0.012	0.949

In addition, we refer to several studies that conducted experiments to validate the reliability of the gene–phenotype associations predicted by CSGDN in our testing dataset. For example, Sun *et al*. [65] demonstrated that the RNA recognition motif (*RRM*, Ghir_A11G026810) positively regulates fiber elongation and fiber length of *G. hirsutum*, and our model accurately predicted that the RRM has a very high positive probability. Furthermore, Zhang *et al*. [66] reported that the growth-regulating factor 5 gene (*GRF5*, Ghir_D13G017310) can also enhance fiber length, and CSGDN successfully predicted this association with a positive probability of 99.8%. These experimental validations further support the performance of our model, which can provide reliable predictions for subsequent experimental research. These external references can show that our predicted gene–phenotype association is consistent with the facts. This not only verifies the accuracy of our model, but also demonstrates the powerful role of the model in predicting gene–phenotype association. We also find that when we used gene sequence similarity as a feature of the model input, the model’s prediction results showed that many genes with higher sequence similarity showed the same association with specific phenotypes. This demonstrates that genes with similar sequences may have similar functional roles or participate in the same biological pathways associated with phenotypes. This observation is consistent with the underlying biological patterns and suggests that genes with high sequence similarity may serve as candidates for further exploration to understand the molecular mechanisms underlying the phenotypes. Furthermore, the excellent performance of our model CSGDN emphasizes the importance of considering sequence similarity as a feature in computational models predicting gene–phenotype associations.

## Conclusion

Association prediction between gene and phenotype plays a crucial role in grasping complex biological and genetic process in crops. We propose a novel CSGDN model to address two major issues, including costs and noise. CSGDN uses the diffusion method to capture the potential associations with minimal sample size. Contrastive learning strategies are utilized to unify the node presentations from two view created by stochastic perturbation. Multiview contrastive loss demonstrates outstanding results in interference and noise.

Extensive experiments show that CSGDN achieves state-of-the-art performance, and outperforms both traditional methods and deep-learning baselines. Case study illustrate the superior performance of our model on crop datasets. CSGDN can predict positive/negative/irrelevant associations between gene and phenotype, and predictions are mostly correct using TWAS results as a reference.

CSGDN significantly addresses the two issues mentioned above and provides highly reliable predictions for further experimental validation. Our model can overcome the considerable cost introduced by the large sample size and reduce various types of noise in the prediction process by enhancing its robustness to interference.

Moreover, it is worth noting that the model is versatile and can accept other types of data input. For instance, researchers engaged in functional genomics can input gene–phenotype associations obtained from CRISPR, RNAi, or overexpression experiments into the CSGDN. This allows the model to provide new candidate genes for researchers in these fields.

However, the current model lacks interpretability, which limits its direct application in crop breeding. In the future, we look forward to applying advanced mechanisms to improve the interpretability to provide clearer insights into underlying gene–phenotype associations.

**Key Points**

- Predicting positive and negative gene–phenotype associations is crucial to understanding complex phenotypes in organisms, particularly in crop breeding. However, traditional methods such as GWAS and TWAS require large sample sizes, making them costly and time-consuming. Furthermore, data noise and experimental errors make accurate predictions difficult, creating the need for more efficient and noise-resistant models.
- To address the challenges of large sample sizes and noise, we propose **CSGDN** (Contrastive Signed Graph Diffusion Network), which uses a signed graph diffusion method to uncover hidden gene–phenotype associations. This diffusion process allows the model to capture complex regulatory relationships even with smaller datasets, significantly reducing the overall cost of data collection and analysis. Additionally, CSGDN uses stochastic perturbation methods to create two different views of both the original and diffused graphs. A multiview contrastive learning framework unifies the node representations from both views, helping the model resist noise and interference introduced by experimental errors, improving the accuracy of predictions.
- We validated CSGDN on three crop datasets: *Gossypium hirsutum*, *Brassica napus*, and *Triticum turgidum*, where it outperformed baseline models, achieving AUC improvements of up to 9.29%. The model demonstrated strong performance even with small sample sizes and under data perturbation, consistently improving key metrics such as F1 and Micro.
- CSGDN provides a robust and cost-effective solution for predicting gene–phenotype associations, addressing the challenges of high cost and noise, and advancing the study of complex phenotypes in crops.
